# Supplementary material for: Structures of Chaetomium thermophilum TOM complexes with bound preproteins
Source: Proc Natl Acad Sci U S A. 2025 Jul 17;122(29):e2507279122. doi: 10.1073/pnas.2507279122 (PMC12305020; doi:10.1073/pnas.2507279122)
Supplement: Supplementary file 1 — Appendix 01 (PDF) [file pnas.2507279122.sapp.pdf]

PNAS

Supporting Information for

Structures of *Chaetomium thermophilum* TOM complexes with bound preproteins

Ahmed-Noor A. Agip[1†*], Pamela Ornelas[1†], Tzu-Jing Yang[2], Ermanno Uboldi[2], Sabine Häder[1], Melanie A. McDowell[2], Werner Kühlbrandt[1*]

[1] Department of Structural Biology, Max Planck Institute of Biophysics, Frankfurt, Germany, 60438
[2] Membrane Protein Biogenesis Research Group, Max Planck Institute of Biophysics, Frankfurt, Germany, 60438

† These two authors contributed equally to this work
*Corresponding authors: Ahmed-Noor A. Agip, Werner Kühlbrandt
Email: agip@biophys.mpg.de, werner.kuehlbrandt@biophys.mpg.de

This PDF file includes:

Figures S1 to S18
Table S1
Legend for Movie S1
SI References

Other supporting materials for this manuscript include the following:

Movie S1

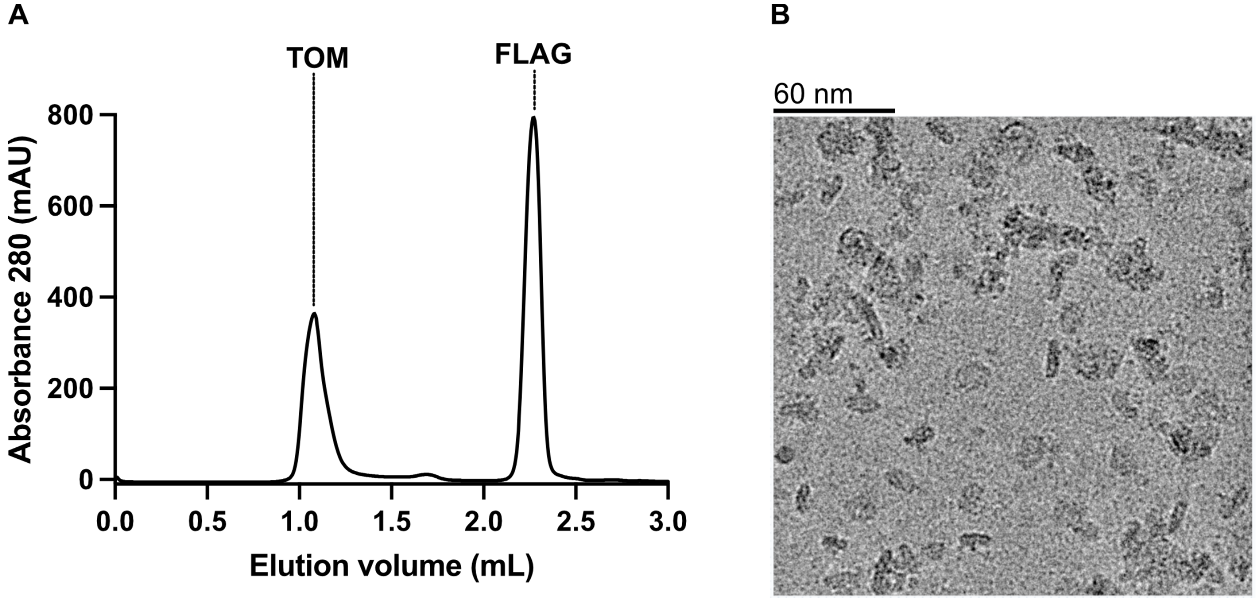

Figure S1. Purification of *Chaetomium thermophilum* TOM from isolated mitochondria. (**A**) Size-exclusion chromatography profile using a Superdex 200 Increase 5/150 GL for the purified TOM complex separated from the FLAG peptides. (**B**) An exemplary cryoEM micrograph with the scale bar above the image.

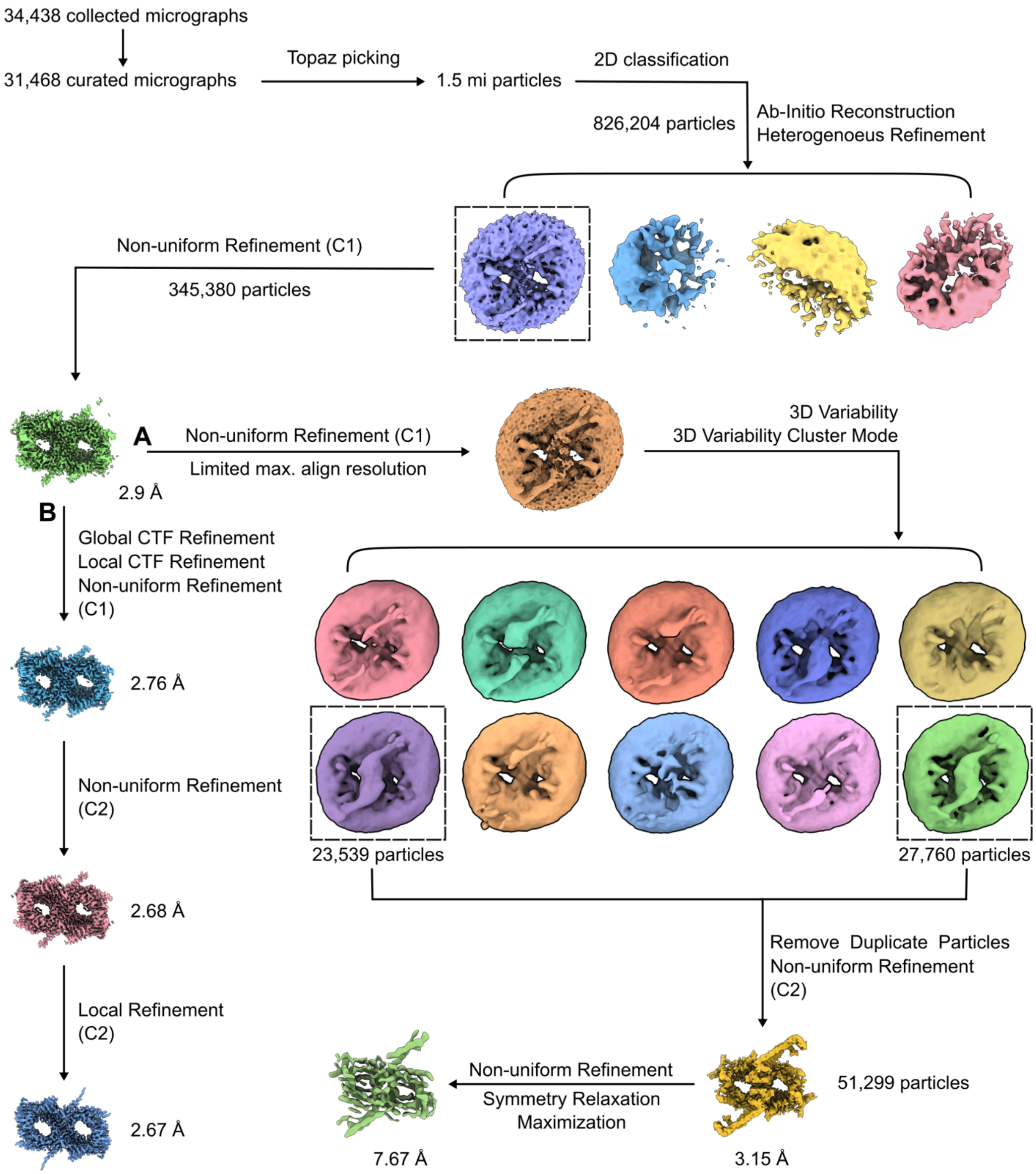

Figure S2. CryoEM processing pipeline for the pALDH bound dataset. (**A**) The workflow that resulted in two Tom20 bound C2-symmetrised and symmetry-relaxed holo complexes at resolutions of 3.15Å and 7.67Å, respectively. (**B**) The processing pathway that yielded the TOM core structure at 2.67Å resolution.

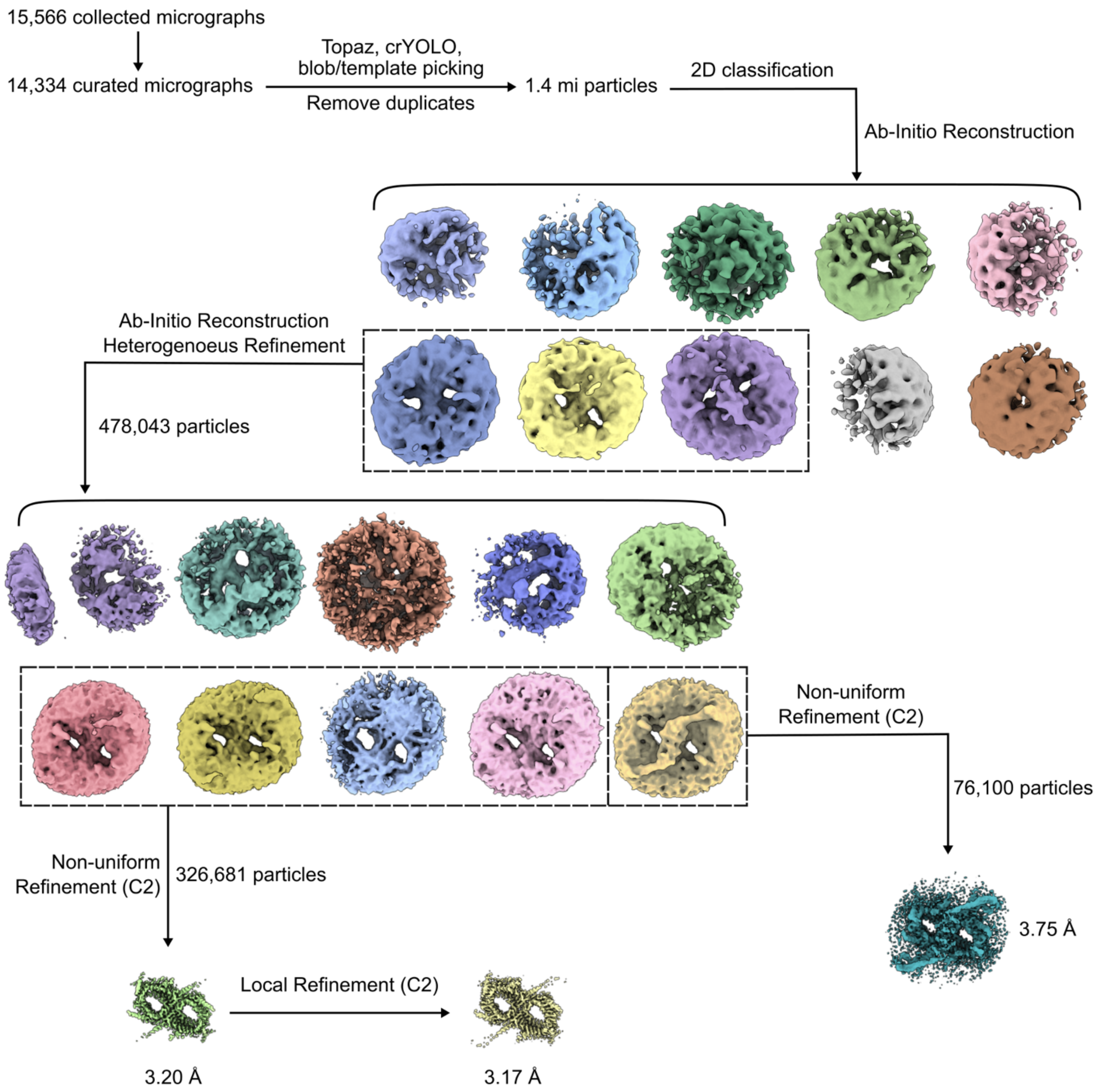

Figure S3. **CryoEM processing pipeline for the substrate-free dataset.** The workflow yielded the C2-symmetrized TOM core and holo complexes at 3.17Å and 3.75Å resolution, respectively.

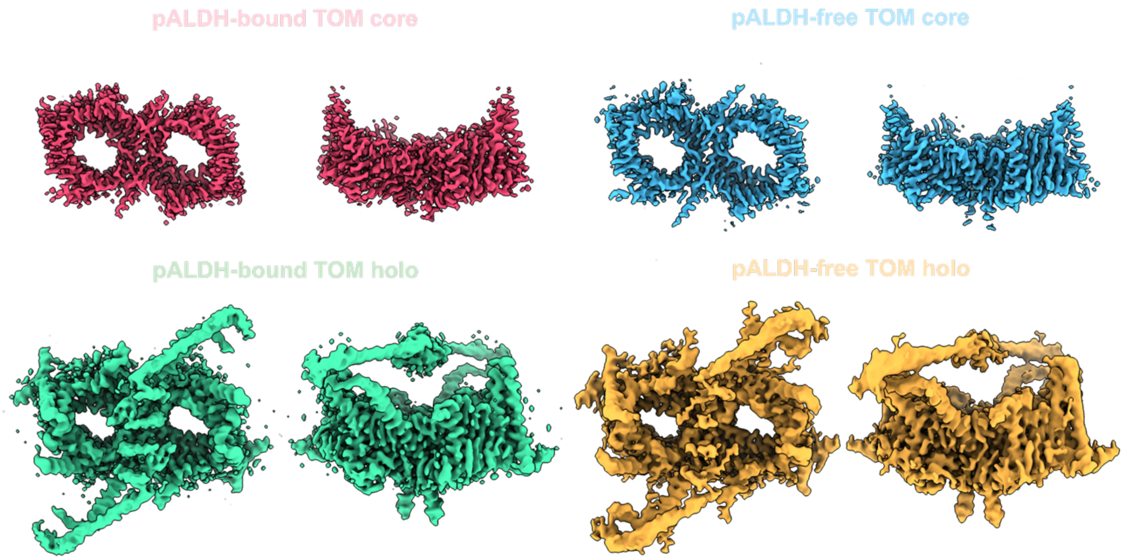

Figure S4. Comparisons of the final cryoEM reconstructions of the *C. thermophilum* TOM complexes. The volumes were viewed in UCSF ChimeraX (1), and the 'hide dust' function was applied only to pALDH-free TOM with a size limit set to 5 Å.

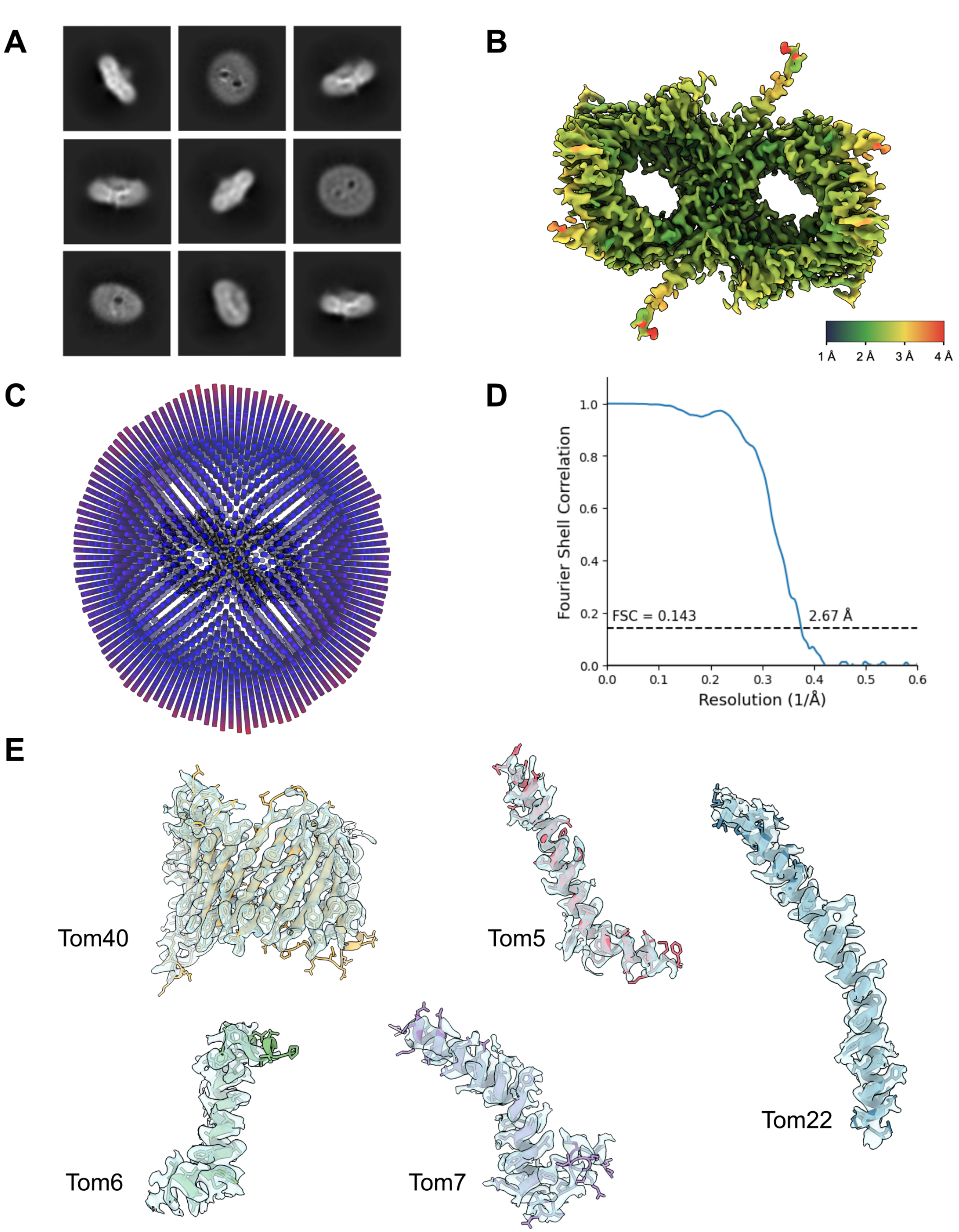

Figure S5. **Quality assessment of the *C. thermophilum* TOM core single-particle dataset.** (**A**) 2D class averages of the TOM holo complex. (**B**) Local resolution estimation of the reconstruction with a scale bar below to indicate resolution values. **C**) The angular distribution of the particles in the dataset is displayed over the volume with the length of the bar indicating

abundance. (**D**) The corrected FSC curve of the reconstruction, where the resolution was determined at a threshold of 0.143. (**E**) The cartoon representation of the subunits of the TOM core complex with their cryoEM densities.

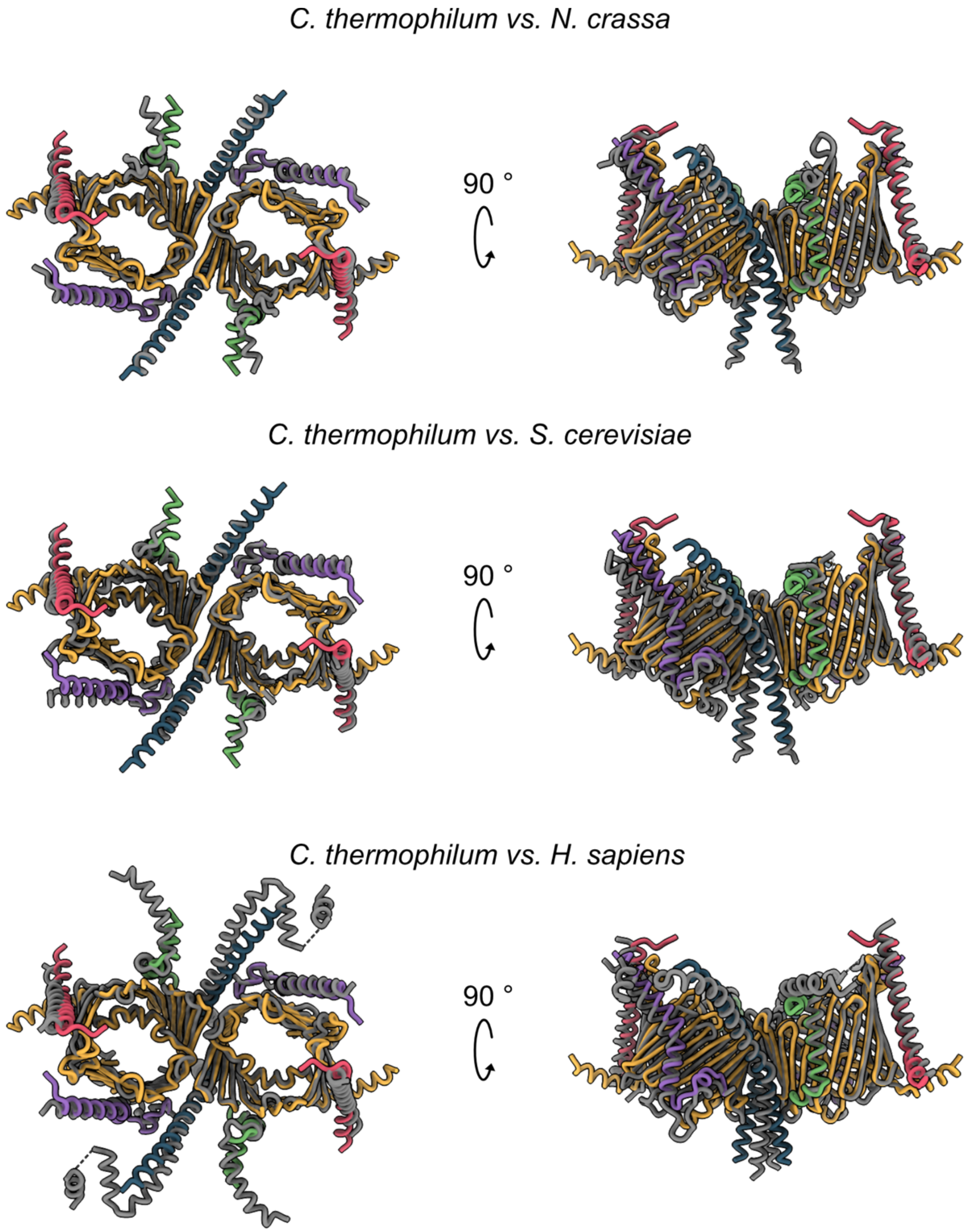

Figure S6. **Structural comparison of the *C. thermophilum* TOM core complex with other species.** Structures from other species (gray) were superposed onto that of *C. thermophilum* (colored) in UCSF ChimeraX and displayed as cartoons (2). The *C. thermophilum* subunits are colored yellow (Tom40), blue (Tom22), purple (Tom7), green (Tom6) and pink (Tom5). The RMSD values between *C. thermophilum* and *N. crassa* (8B4I), *S. cerevisiae* (6UCU), and *H. sapiens* (7CP9) were 0.725 Å (based on 286 atoms), 0.812 Å (based on 268 atoms), and 0.840 Å (based on 214 atoms).

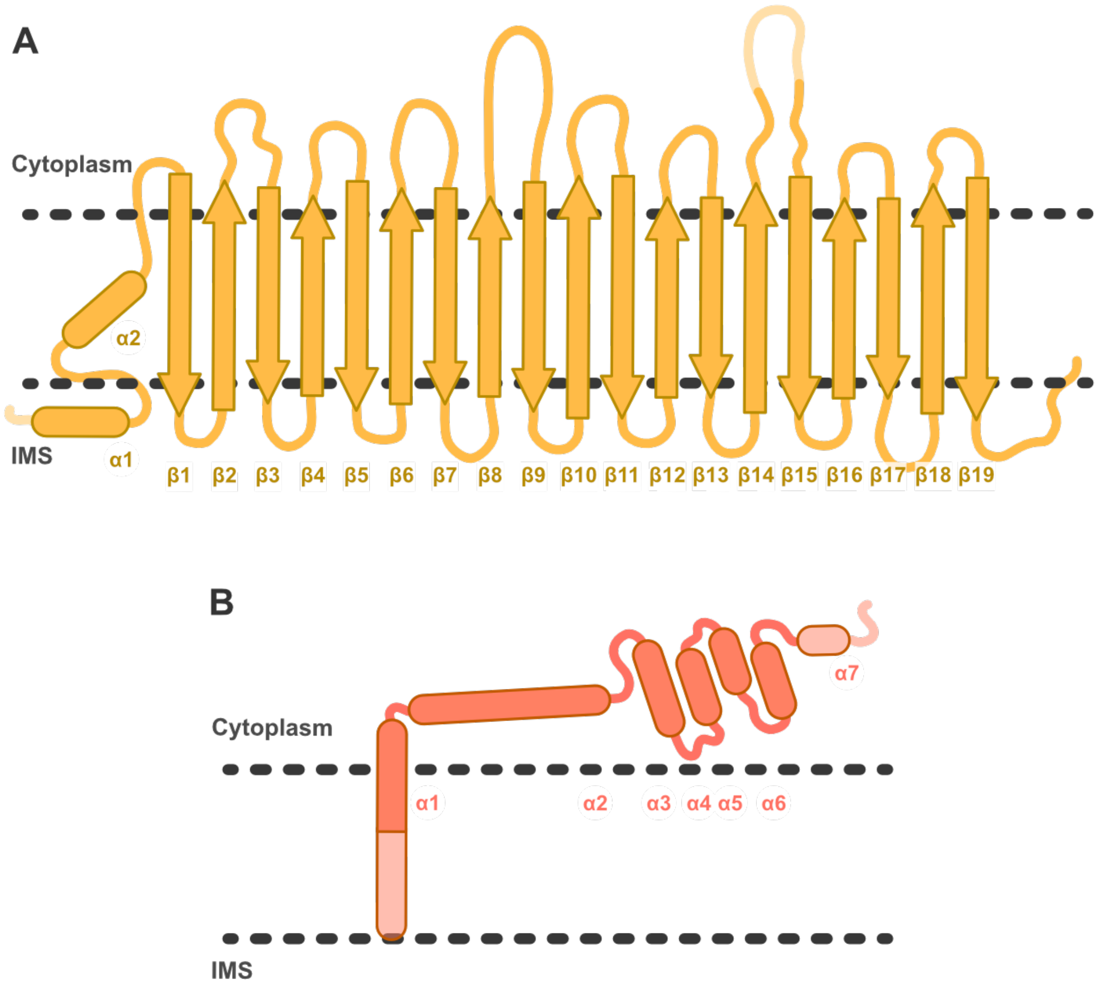

Figure S7. The topology of the *C. thermophilum* Tom40 and Tom20 subunits. (**A**) A schematic representation of the nineteen β-strands of Tom40, the interconnecting loops and the two α-helices. (**B**) A schematic representation of the seven α-helices of Tom20, including the tetratricopeptide repeat (TPR) fold α3-6. Unresolved regions are shown in a lighter color.

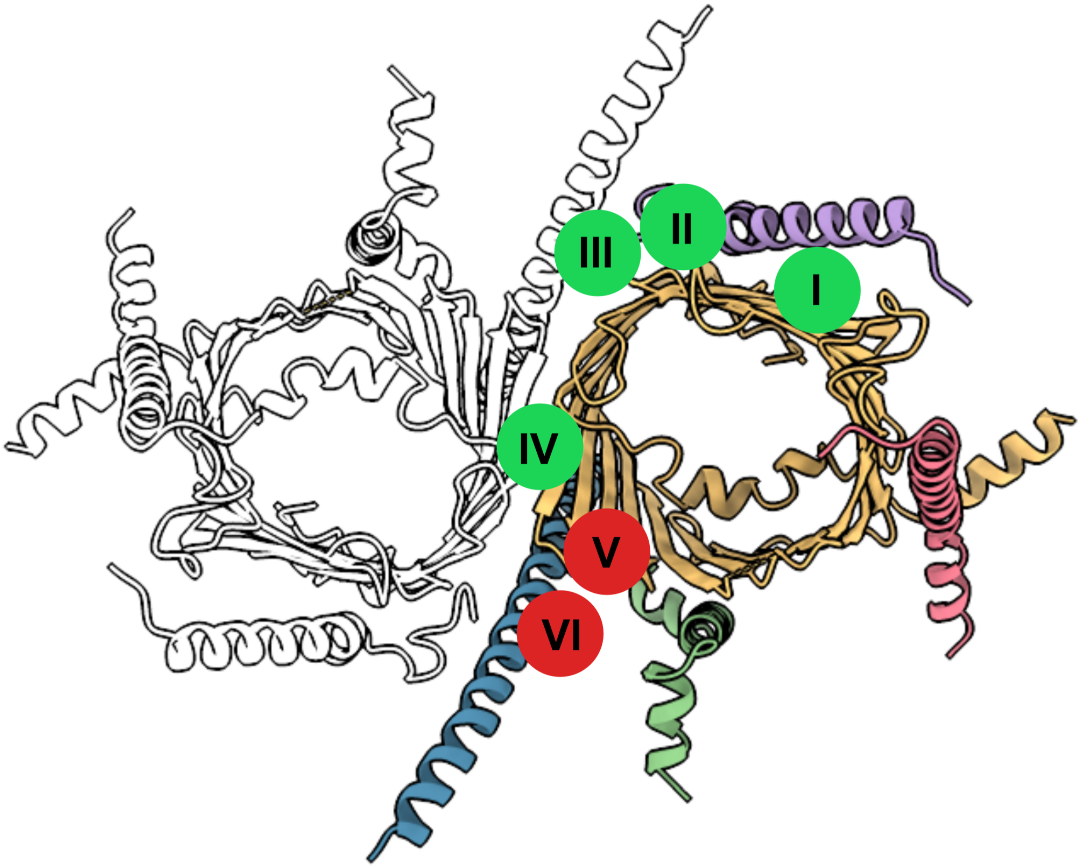

Figure S8. Conservation of lipid positions across organisms. The *C. thermophilum* TOM complex is represented in cartoon with only one protomer colored. Lipid positions in *C. thermophilum* (numbered) are compared with those of *N. crassa* (PDB: 8B4I) and humans (PDB: 7CP9). Green and red circles indicate whether those positions are conserved or not, respectively.

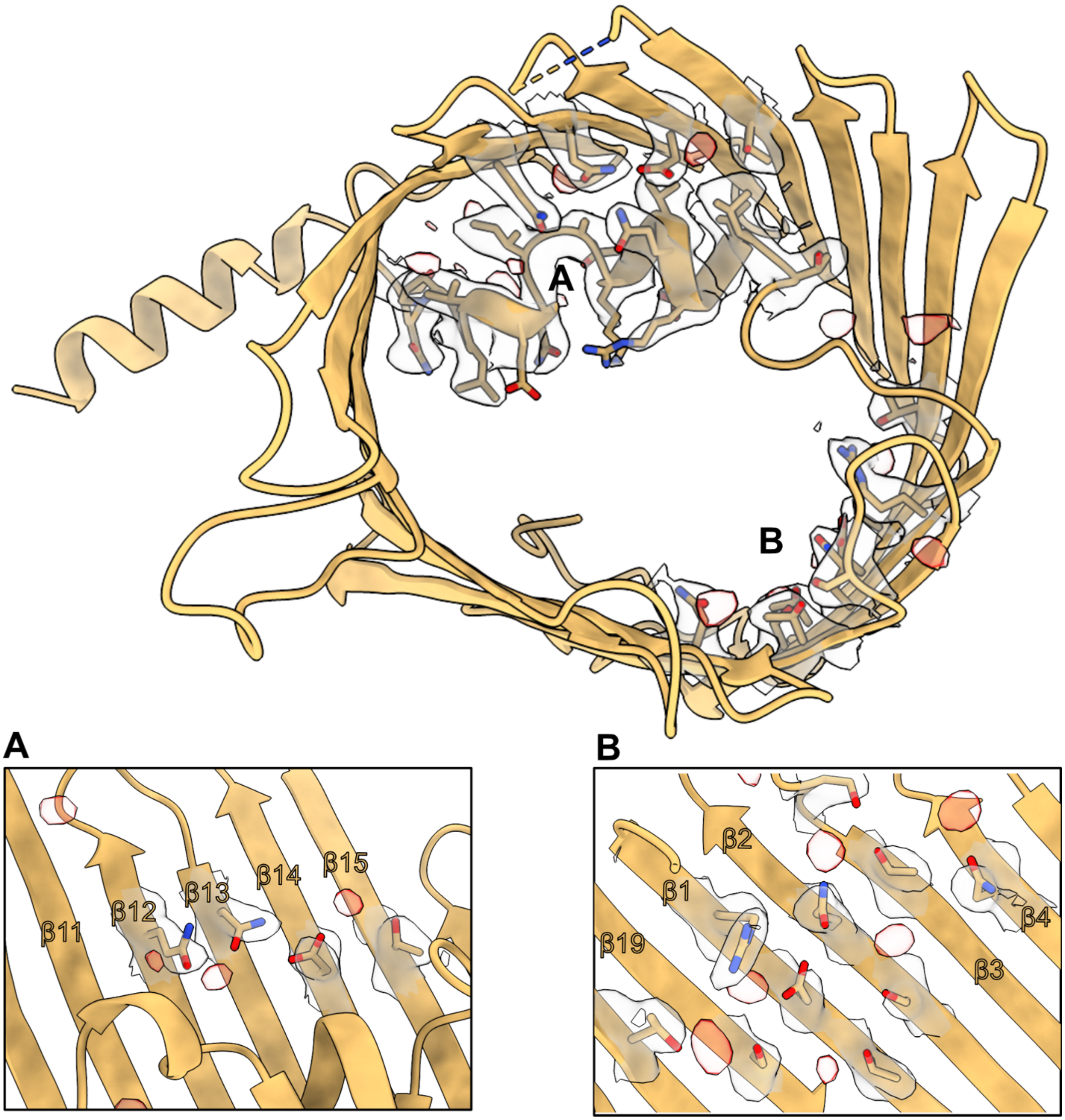

Figure S9. Clusters of unassigned waters or ions. (Top) Densities of suspected waters or ions (red) observed within the Tom40 pore from the cytoplasmic side. (**A**-**B**) Close-up of amino acid residues adjacent to the unassigned densities.

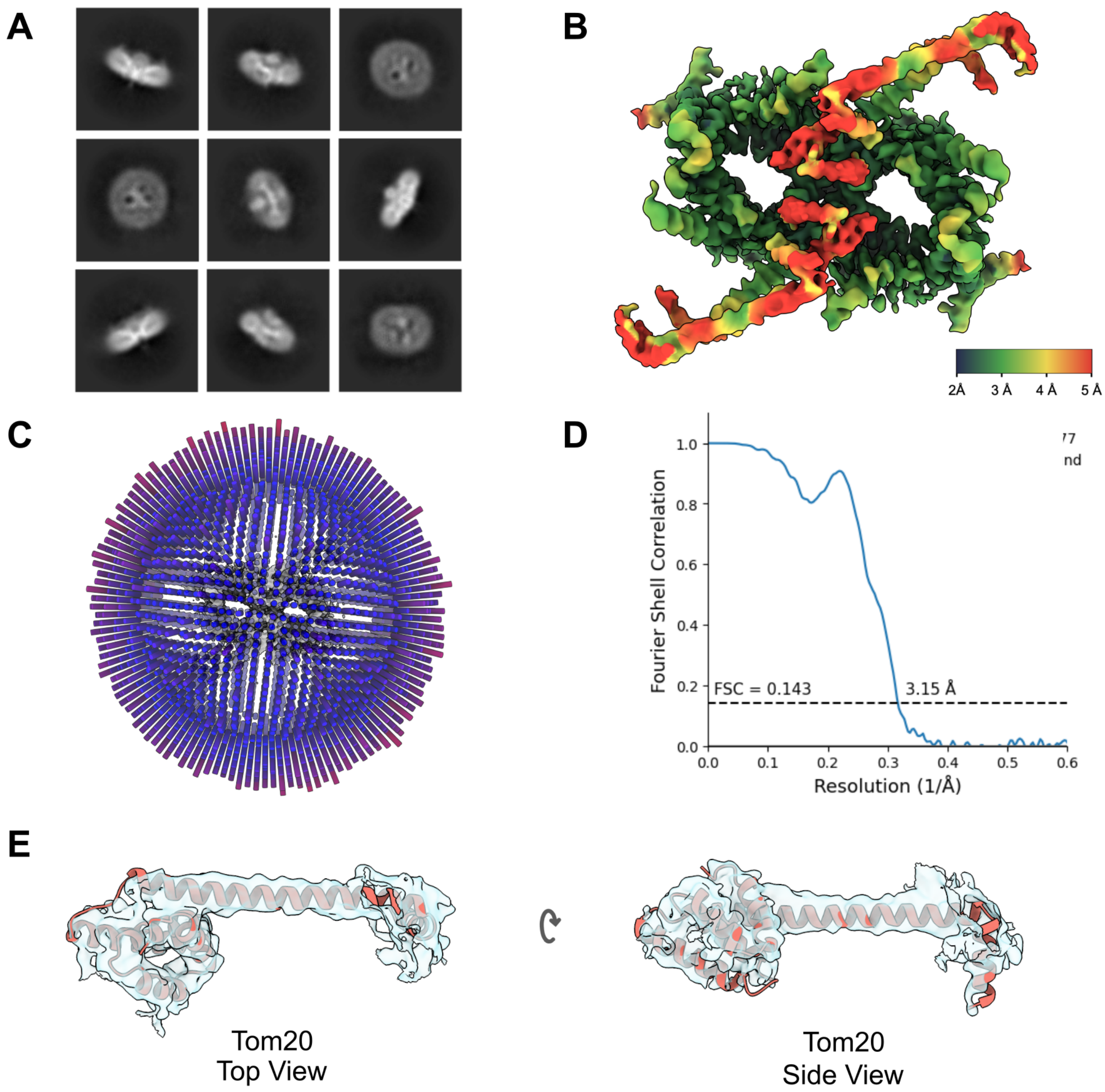

Figure S10. Quality assessment of the *C. thermophilum* TOM holo single-particle dataset. (**A**) 2D class averages of the TOM holo complex. (**B**) Local resolution estimation of the reconstruction with a scale bar below to indicate resolution values. (**C**) The angular distribution of the particles in the dataset is displayed over the volume, with the length of the bar indicating abundance. (**D**) The FSC curve of the reconstruction, where the resolution was determined at a threshold of 0.143. (**E**) The cytoplasmic portion of the Tom20 subunit in cartoon representation with its cryoEM density.

A

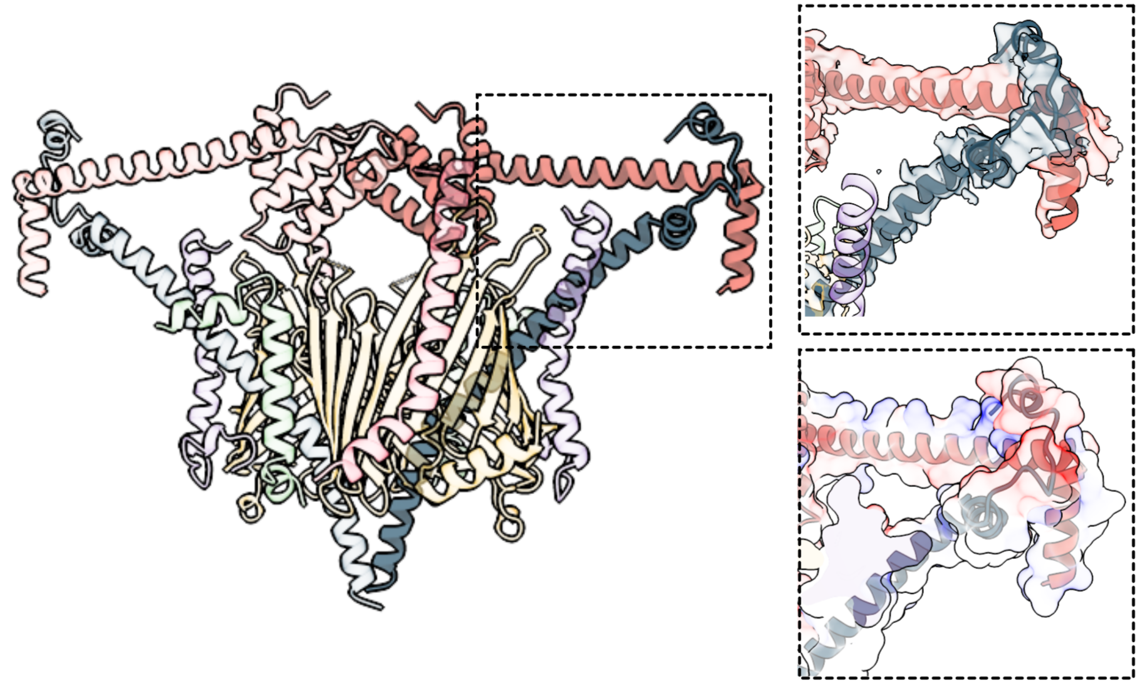

B

Tom22

Tom20 contact site

```
                10        20        30        40        50        60        70
Chaetomium     -----MVQLVEVEDEHFTQPQPG-------PEEDD-----DEY-TDTDSEISTESNFDPSEETLADRLHALRDMVPP
Neurospora     -----MVQLTEVEDEHFQQPQVG-------PEEDD-----EDF-TDTDSEISVDSDYES-QETFTDRLYALRDMVSP
Saccharomyces  -----MVELTEIKDDVVQLDEPQFSRNQAIVEEKASATNNDVVDDEDDSDSDFEDEFD-ENETLLDRIVALKDIVPP
Homo           ------------MAAAVAA---AGAGE-PQSPDELLPKG--D---AEKPEEELEEDDDEELDETLSERLWGLTEMFPE
Drosophila     MDSDPEIEFIEKDSGMSS---LGGSK-DETPERRAVA--------ATSNDPQRENYDDEPDETASERFWGLTEMFPE

               80        90        100       110       120       130       140       150
Chaetomium     AYRGWIYHKYEQTTSAVRKALSFAGRAAWTVSVTALLVGVPFSLAYGEDQQYAAMEQEQRMRELGGEVLTAGAPGSQ
Neurospora     TTRGWFYHKYSTTTNFVKSTLSFAGRAAWAVSVSGLLIGVPFAIAFAEDQNYAAMEQEARMRELGSDVLTAGGEGQA
Saccharomyces  GKRQTISNFFGFTSSFVRNAFTKSGNLAWTLTTTALLLGVPLSLSILAEQQLIEMEKTFDLQSDANNILAQGEKDAA
Homo           RVRSAAGATFDLSLFVAQKMYRFSRAALWIGTTSFMILVLPVVFET----EKLQMEQQQQLQQR--QILLGPNTGLS
Drosophila     PVRNAVGAVSSATVKSVKGFYSFSCNASWIFFTSAVILFAPVIFET----ERAQMEELHKSQQK--QVLLGPGSAMG

               160       170       180
Chaetomium     GGGLTAEQVNAALGRSEAKPAL----
Neurospora     GT---AEKTLAAIGGEGARPAL----
Saccharomyces  ATAN----------------------
Homo           G------------GMPGALPSLPGKI
Drosophila     P------------GGPSP--SLPLIR
```

Tom20

Tom22 contact site

```
                10        20        30        40        50        60        70
Chaetomium     MS-SSPSP-AIVATAAVATLAAGVLAYAAYFDYQRRHNAEFRRQLRRNERRQARAEKDLAEASAKAQRQRIK
Neurospora     ----MPSQAVTYTTAAVAAVATGFLAYAVYFDYKRRNDPEFRRQLRRSARRQARQEKEYAELSQQAQRQRIR
Saccharomyces  MSQSNPILRGLAITT--AIAALSATGYAIYFDYQRRNSPQFRKVLRQRAKEQAKMEEQAKTHAKEVKLQKVT
Homo           -------MVGRNSAIAAGVCGALFIGYCIYFDRKRRSDPNFKNRLRERRKKQKLAKERAGL-----------
Drosophila     ----MIEMNKTAIGIAAGVAGTLFIGYCIYFDKKRRSDPEYKKKVRERRRRNKK--TGTAK-----------

               80        90        100       110       120       130       140
Chaetomium     QA-VDEAKEEGFPTSAEDKEAFFLEQVQAGEMMSADPSKHLEAALCFYKALKVYPTPGDLINIYDKTVSKPI
Neurospora     QM-VDEAKEEGFPTTSDEKEAYFLEQVQAGEILGQDPTKAIDASLAFYKALKVYPTPGDLISIYDKTVAKPI
Saccharomyces  EFLSMELAKDPIPSDPSEREATFTTNVENGERLSMQQGKELEAASKFYKALTVYPQPADLLGIYQRSIPEAI
Homo           -------SKLPDLKDAEAVQKFFLEEIQLGEELLAQGEYE-KGVDHLTNAIAVCGQPQQLLQVLQQTLPPPV
Drosophila     -------SGVPNLNDHEAIERYFLQEIQLGETLIARGDFE-SGVEHLANAIVVCGQPARLLQVLQSSLPAQV

               150       160       170       180       190
Chaetomium     LDILAEMIAYDSSLRIGTAYTGPA--GVDVAD-LMR--EM-GAVPGVGLD---
Neurospora     LDILAEMIAYDPSLKIGTNYTG----GVDVAE-LMR--EM-ASAPGVGLD---
Saccharomyces  YEYIILMIAILPPANVASFVKGVV--GSKAESDAVA--E------ANDIDD--
Homo           FQMLLTKLPTISQ-RIVSAQSLA-------EDDVE------------------
Drosophila     FAMLIVKMQEFGN-RAAEGNDGPIVLGQSSEQQLDGAKIIECSSGNASIDDLE
```

Figure S11. **Molecular interactions of Tom20 with Tom22.** (**A**) The TOM holo complex is depicted as a cartoon representation with one of the Tom22 (blue) and Tom20 (red) displayed as opaque. The dashed boxes highlight the interaction between the two subunits, with the close-up showing their corresponding cryoEM density (top) or the model-derived coulombic electrostatic potential (bottom). (**B**) Sequence alignments of Tom22 and Tom20 across various organisms. Conserved residues for the Tom22 and Tom20 contact sites are highlighted in red and blue, respectively.

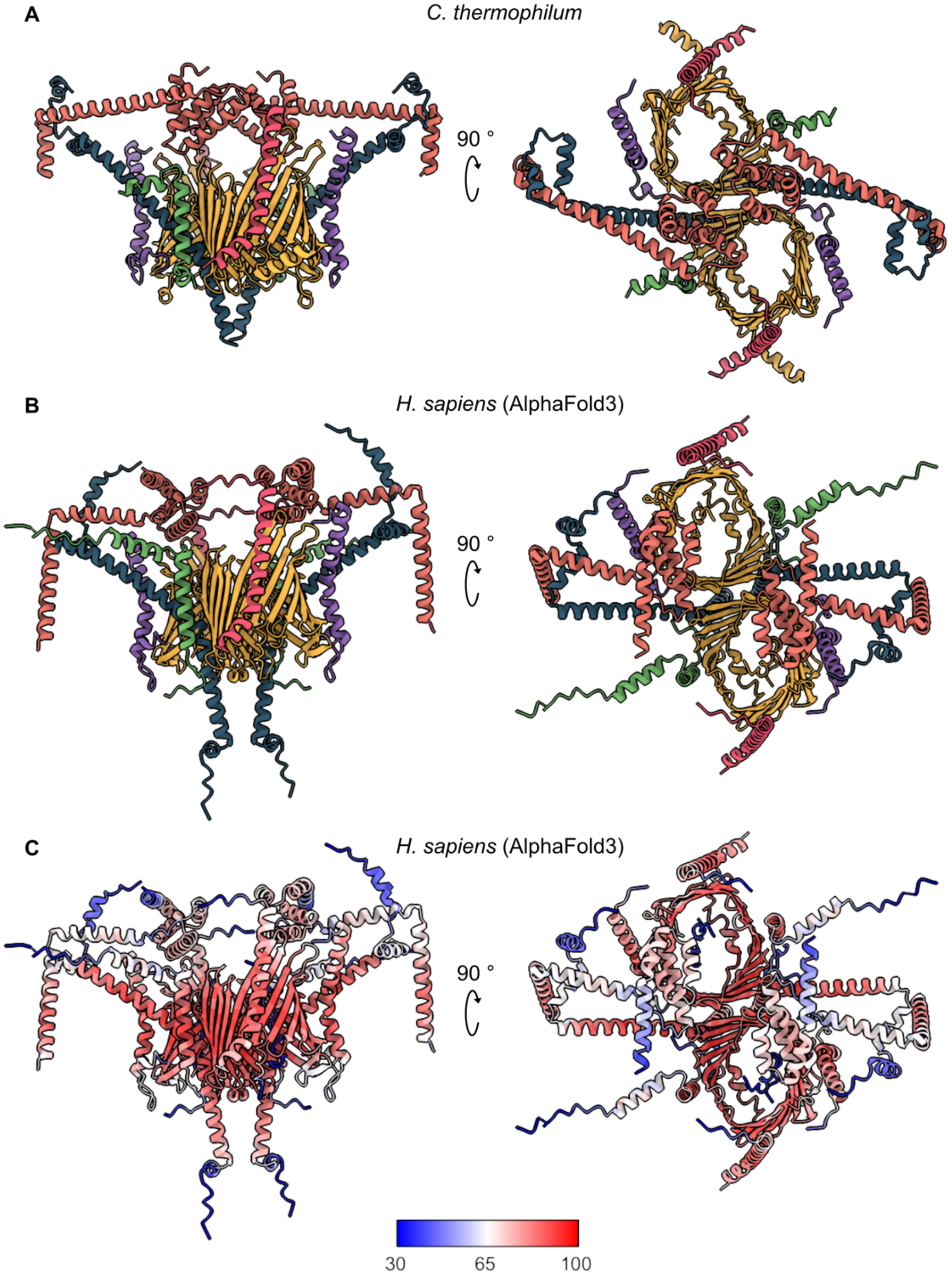

Figure S12. **The symmetrical TOM holo complex is not unique to *C. thermophilum***. A visual comparison between the *C. thermophilum* (**A**) and the AlphaFold3 (2) predicted TOM holo complex from *H. sapiens* (**B**). (**C**) The predicted local distance difference test (pLDDT) is represented on the model with a corresponding scale bar to indicate its value.

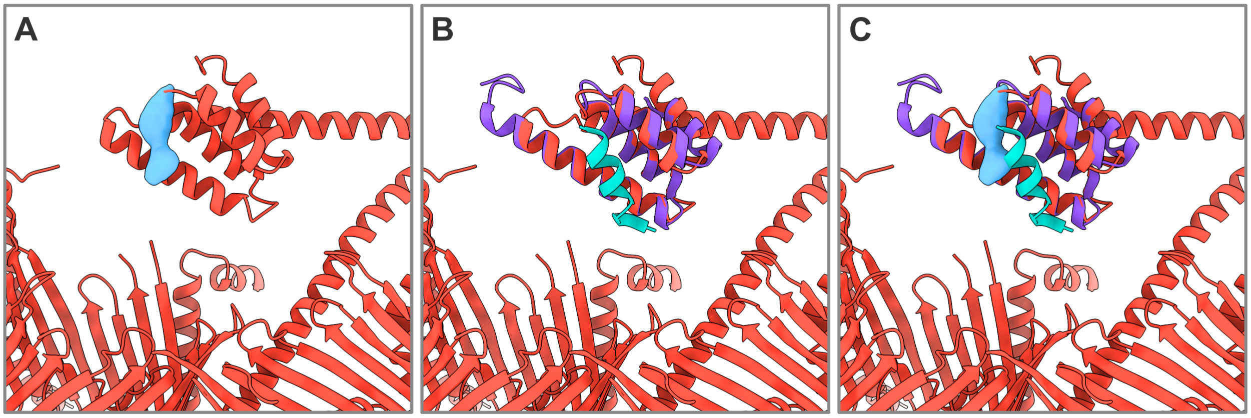

Figure S13. **Tom20 TPR domain and receptor-preprotein interaction**. (**A**) A bound preprotein density (blue) is shown bound to the *C. thermophilum* Tom20 structure (red). (**B**) The rat crystal structure of Tom20 (purple, PDB: 3AWR) with bound pALDH (cyan) is superposed to the *C. thermophilum* structure. (**C**) An overlay of (**A**) and (**B**).

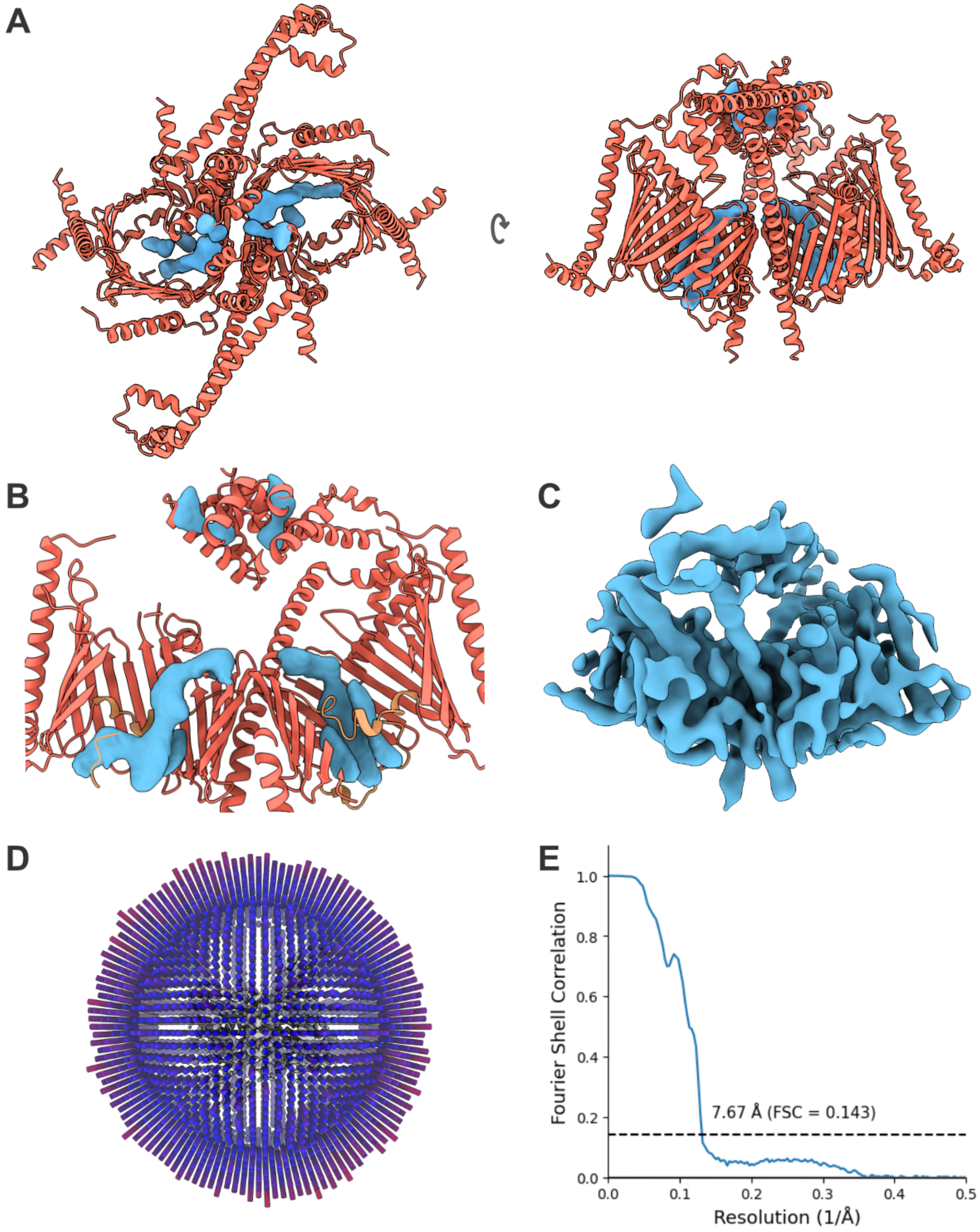

Figure S14. **Preprotein binding to the TOM holo complex.** (**A**) Preprotein densities displayed alongside the TOM holo model were generated by subtraction of the unbound TOM holo map from the bound map using UCSF ChimeraX (2). (**B**) A close-up and sectional view of preprotein densities. (**C**) Non-subtracted cryoEM density of the pALDH-bound TOM holo complex. (**D**)

Angular distribution of particles from the symmetry relaxed dataset (**Figure S2**). (**E**) The FSC curve of the reconstruction, where the resolution was determined at a threshold value of 0.143.

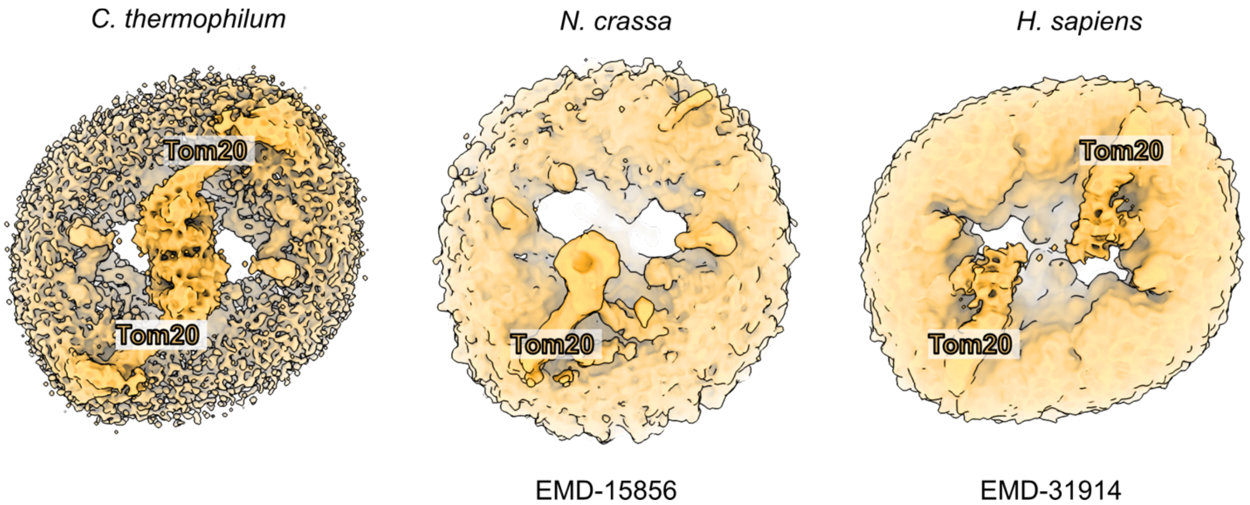

Figure S15. Comparison of cryoEM maps of the TOM holo complex across species. Contour levels for each cryoEM reconstruction were adjusted in UCSF ChimeraX to ensure that Tom20 (labeled) would be visible (1).

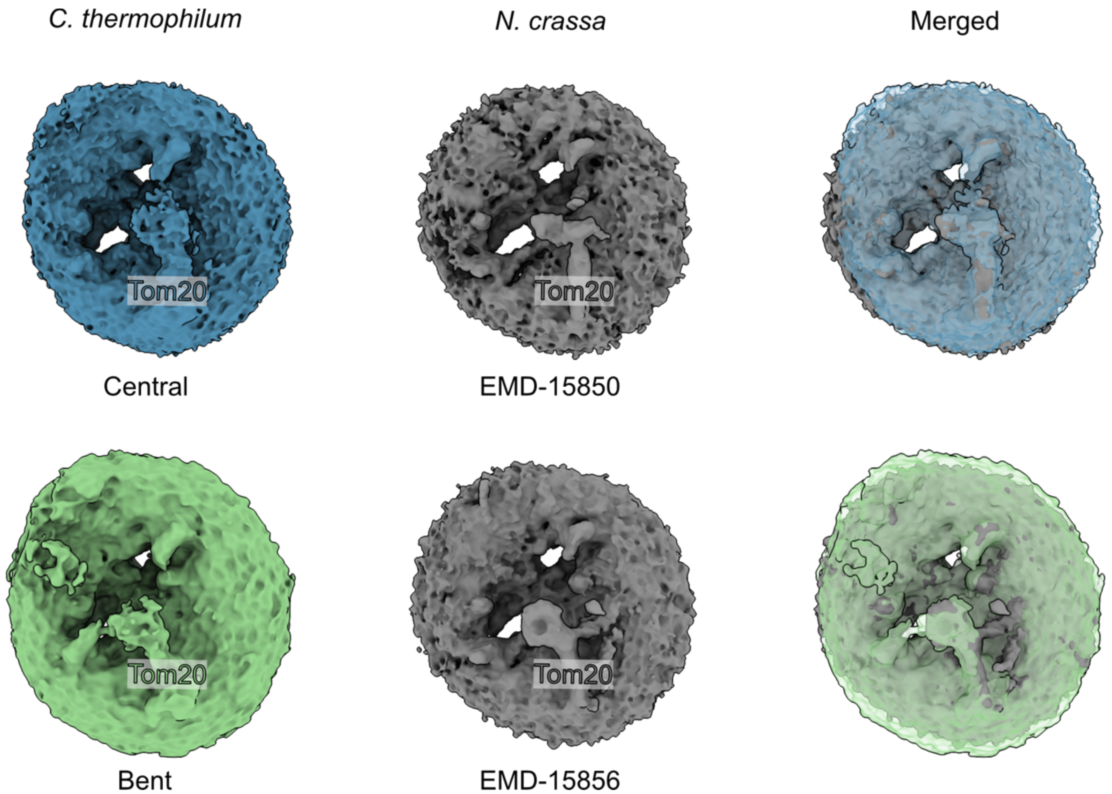

Figure S16. Conformational differences of Tom20 between *C. thermophilum* and *N. crassa*. Two conformations from *C. thermophilum* (Figure 4) are compared side-by-side and superposed to that of *N. crassa*. The contour level for each cryoEM map was set in UCSF Chimerax so that Tom20 (labeled) would be visible (1).

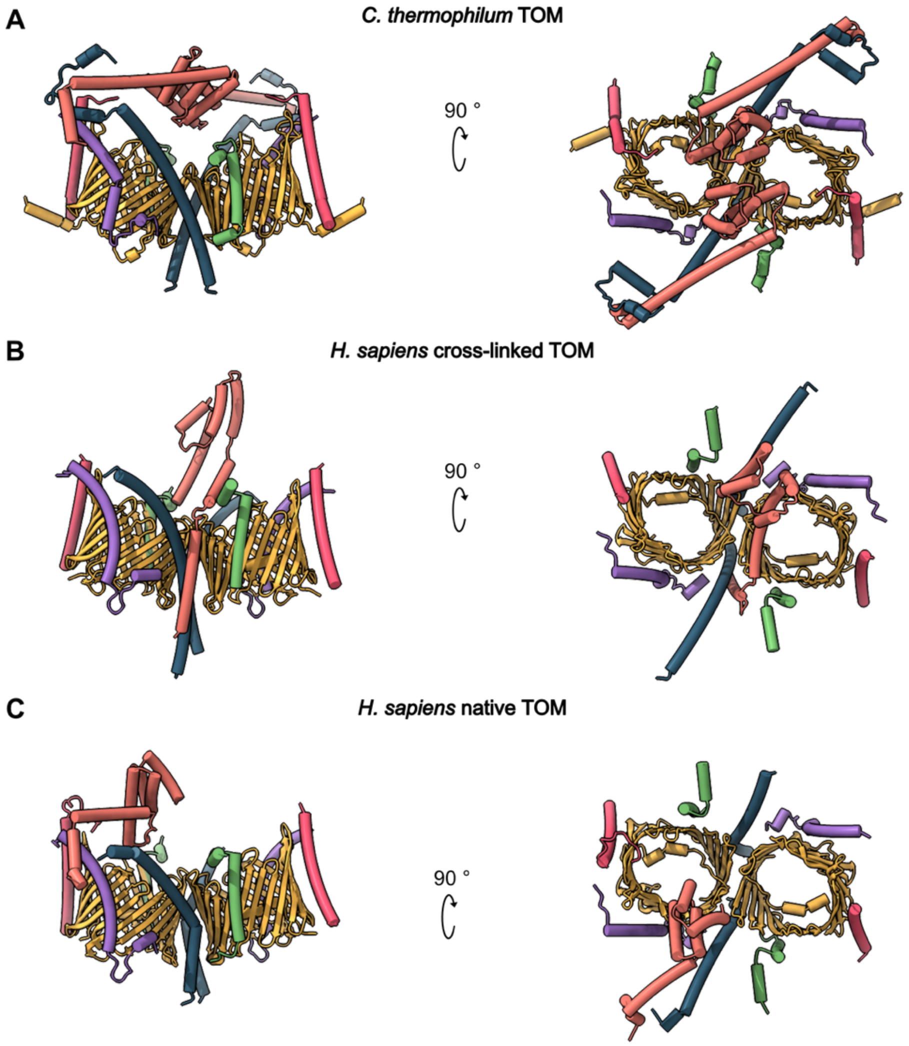

Figure S17. Positions of Tom20 in *H. sapiens* compared to *C. thermophilum.* Our *C. thermophilum* TOM holo complex (**A**) compared to the cross-linked (**B**) human TOM (PDB: 8XVA) and (**C**) TOM subunits in the human TOM-PINK1-VDAC complex (PDB: 9EIH). The colours of TOM holo subunits are: Tom40, yellow; Tom22, blue; Tom20, orange; Tom5, Tom6 and Tom7, red, green and purple.

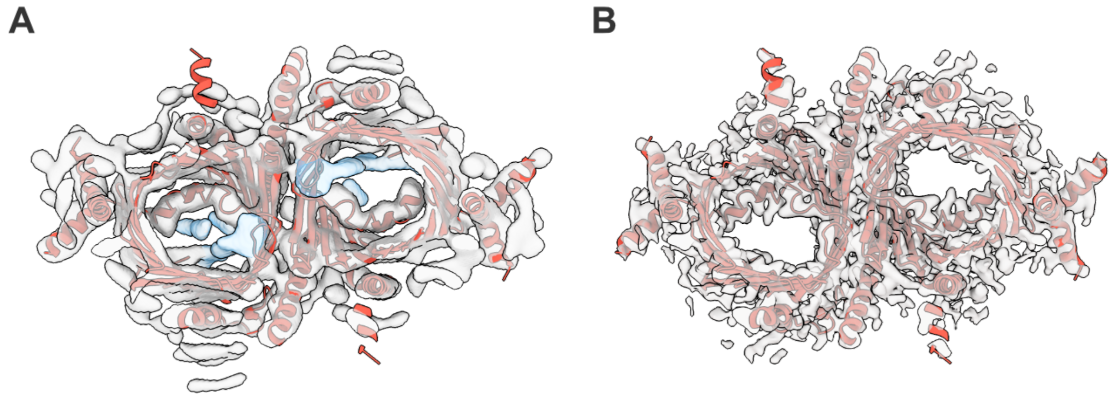

Figure S18. Visualization of preprotein densities in the TOM complex. Close-up and sectional view from the cytoplasmic side of the (**A**) symmetry-relaxed map of the preprotein-bound TOM holo complex and (**B**) the unbound TOM holo complex. The atomic model is shown in orange. The absence of corresponding preprotein densities (blue in **A**) in panel **B** confirms that it is specific to the bound state and cannot be explained by the TOM complex model alone.

Table S1. CryoEM data collection, refinement and validation of the *C. thermophilum* TOM core complex.

	Preprotein-bound TOM core	Preprotein-bound TOM holo	Preprotein-free TOM core	Preprotein-free TOM holo
**Data collection and processing**				
Magnification	215,000x	215,000x	105,000x	105,000x
Voltage (kV)	300 kV	300 kV	300 kV	300 kV
Electron exposure	70 $e^-/Å^2$	70 $e^-/Å^2$	60 $e^-/Å^2$	60 $e^-/Å^2$
Defocus Range (µm)	-1.6 to -2.4	-1.6 to -2.4	-0.9 to -2.4	-0.9 to -2.4
Pixel size (Å)	0.573 Å	0.573 Å	0.837 Å	0.837 Å
Symmetry imposed	C2	C2	C2	C2
Initial particles	1,443,703	1,443,703	1,379,719	1,379,719
Final particles	345,380	51,299	326,681	76,100
Map resolution (Å)	2.7 Å	3.2 Å	3.8 Å	3.2 Å
FSC Threshold	0.143	0.143	0.143	0.143
**Refinement**				
Initial model used	ModelAngelo	AlphaFold3	ModelAngelo	AlphaFold3
Model resolution	2.7 Å	3.2 Å	3.2 Å	3.8 Å
FSC Threshold	0.143	0.143	0.143	0.143
Map sharpening B factor ($Å^2$)	95.0	72.0	141.9	152.4
**Model composition**				
Nonhydrogen atoms modelled	8,644	11,338	8,644	11,338
Total protein residues	1,420	1,790	1,420	1,790
Protein residues modelled	1,020	1,358	1,020	1,358
Percentage of modelled residues (%)	72	76	72	76
Ligands	19	19	19	19
**RMS deviations**				
Bond lengths (Å)	0.007	0.006	0.006	0.006
Bond angles (˚)	0.857	0.739	0.790	0.803
**Validation**				
MolProbity score	1.35	1.21	1.28	1.27
Clashscore	6.28	4.34	5.19	5.13
Rotamer outliers (%)	0.00	0.54	0.24	0.18
**Ramachandran plot**				
Favored (%)	99.00	98.80	98.39	98.50
Allowed (%)	1.00	1.20	1.61	1.35
Disallowed (%)	0.00	0.00	0.00	0.15

Movie S1. Morph video of Tom20. The video generated through UCSF ChimeraX shows the continuous trajectory of Tom20 (red) as it transitions from a bent to a central to an extended conformation (1). The cryoEM volumes depicted are colored according to Figure 4, and the TOM core subunits are shown in gray.

SI References

1. E. F. Pettersen *et al.*, UCSF ChimeraX: Structure visualization for researchers, educators, and developers. *Protein Sci* **30**, 70-82 (2021).
2. J. Abramson *et al.*, Addendum: Accurate structure prediction of biomolecular interactions with AlphaFold 3. *Nature* **636**, E4 (2024).
